# Supplementary material for: Short-Term Exposure Effects of the Environmental Endocrine Disruptor Benzo(a)Pyrene on Thyroid Axis Function in Zebrafish
Source: Int J Mol Sci. 2022 May 23;23(10):5833. doi: 10.3390/ijms23105833 (PMC9148134; doi:10.3390/ijms23105833)
Supplement: Supplementary file 1 [file ijms-23-05833-s001.zip › ijms-1726147-supplementary.pdf]

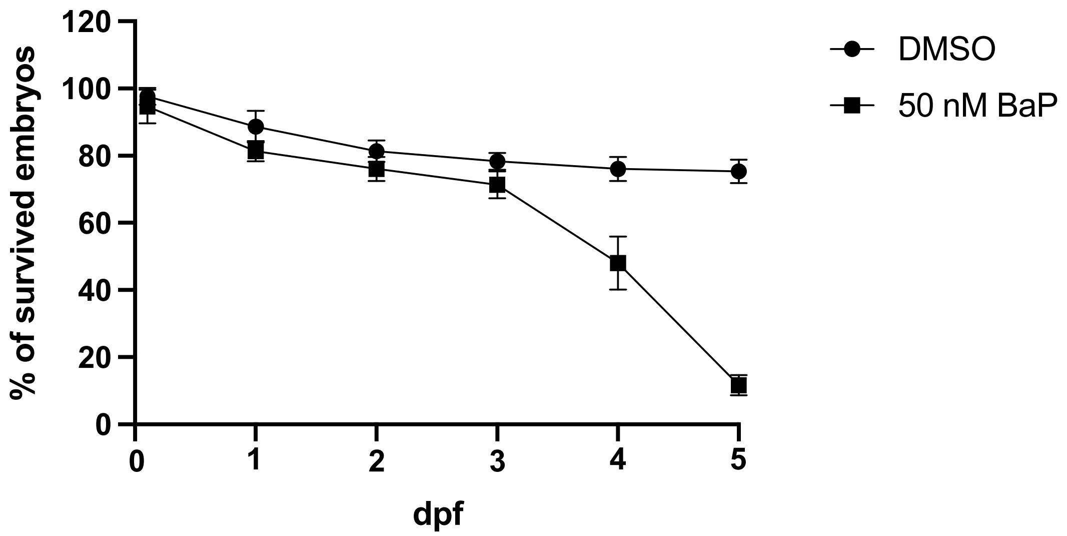

Supplementary Figure S1: Survival rates of 1.2% DMSO controls and 50 nM BaP treated zygotes during embryonic development and larval transition at different stages (dpf = days-post fertilization). Results are reported as Mean % ± SD of live embryos/larvae derived from three independent experiments, using with at least 150 zygotes each.

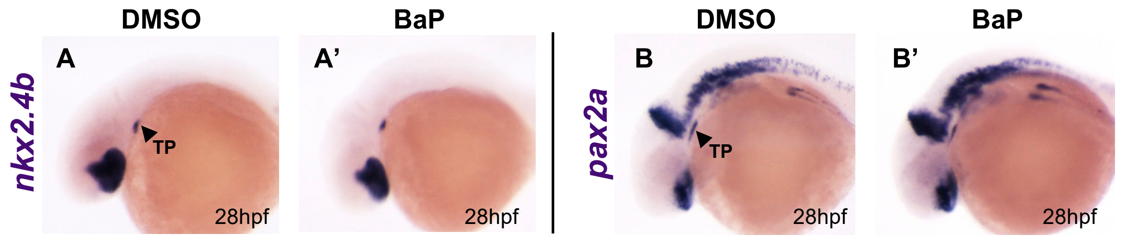

Supplementary Figure S2: WISH of *nxk2.4b* (A and A') and *pax2a* (B and B') at the levels of thyroid primordium (TP) of control and BaP treated embryos at 28 hpf. WISH was performed in duplicate using 30 embryos each.
